# Low Birth Weight Newborns at Public Hospitals in the Awi Zone of Northwest Ethiopia: Birth Defects and Associated Variables

**DOI:** 10.1155/ijpe/2312755

**Published:** 2026-08-08

**Authors:** Tsegaye Mehare, Molla Getie, Getaneh Tegegne, Yewbmirt Sharew

**Affiliations:** ^1^ Department of Biomedical Science, College of Medicine and Health Sciences, Injibara University, Injibara, Ethiopia, inu.edu.et; ^2^ Department of Medical Laboratory Science, College of Medicine and Health Sciences, Injibara University, Injibara, Ethiopia, inu.edu.et; ^3^ Department of Midwifery, College of Medicine and Health Sciences, Injibara University, Injibara, Ethiopia, inu.edu.et

**Keywords:** birth defect, Ethiopia, low birth weight, newborns, prevalence

## Abstract

**Introduction:**

Birth defect is a leading contributor to infant mortality and has significant effects on life expectancy and quality of life. To parents alongside clinical specialists, assessing plus managing infants exhibiting single or multiple anomalies remains remarkably arduous. This study is aimed at assessing the frequency of congenital malformation and associated factors among low birth weight neonates born in public hospitals of Awi Zone, Northwest Ethiopia in 2023.

**Methods:**

We conducted an institutional based cross‐sectional study among 311 mothers of low birth weight newborns. The study was carried out at three primary hospitals (Chagni, Dangla, and Gimjja Bete) and Injibara general hospital from December 2022 to July 2023. Participants included mothers whose LBW newborns were delivered at these facilities, as well as those who delivered at home but presented to the same healthcare settings for care within 24 h of delivery.

**Results:**

A birth defect was observed in 53 (17%) of the newborns. Independent predictors of birth defects in LBW infants included MUAC < 22 cm (AOR: 3.67; 95% CI: 1.52, 8.83), maternal age 36–49 years (AOR: 3.60; 95% CI: 1.26, 10.56), and nonadherence to foliate supplementation before and during early gestation (AOR: 4.46; 95% CI: 1.61, 12.40). In contrast, history of obstetric complications, although yielding an AOR of 1.90, was not significantly associated (95% CI: 0.87–4.15).

**Conclusions:**

In the Awi Zone, 17% of LBW newborns presented with birth defects, with the musculoskeletal system being the most frequently affected site. Significant independent risk factors included failure to take folate supplements before and during early pregnancy, MUAC < 22 cm, and maternal age 36–49 years. Although a history of obstetric complications showed a positive association, it was not statistically significant in the adjusted model.

## 1. Introduction

Birth defects (BDs) are structural or functional deviations that originate during prenatal development [1]. Although they may be diagnosed before birth, at delivery, or well into childhood, they remain primary drivers of pediatric hospitalization and mortality [2–4]. Severe cases impose a considerable physical, financial, and emotional burden affecting healthcare systems and society overall [5, 6]. Although the prevalence of BDs varies depending on the study population and methodology used, it typically ranges from 23.9 to 35.0 per 1000 births [7, 8].

According to WHO, BDs account for 17%–42% of infant mortality globally, even as overall newborn death rates from these causes have declined [9]. However, the absolute burden remains massive, with 7.9 million infants born each year with serious structural or functional defects, a figure that underscores the ongoing urgency of prevention, early detection, and comprehensive care [10].

Low birth weight (LBW) is associated with a higher risk of both congenital defects and perinatal mortality [11, 12]. Many studies have confirmed this association. For example, newborns with cardiac defects including dextro‐transposition of the great arteries, teratology of Fallot, hypoplastic left heart syndrome, and coarctation of the aorta show abnormal fetal growth patterns, particularly affecting birth weight [13]. A study conducted in Mexico reported that preterm newborns with low or very low birth weight (VLBW) had a higher frequency of BDs, primarily affecting the digestive system, genital organs, and musculoskeletal system [14]. Similarly, a study in the United Kingdom found that between 10% and 20% of stillborn and live born infants weighing less than 2000 g at birth had BDs [15, 16].

The Awi Zone in Northwest Ethiopia experiences a high prevalence of LBW and BDs. This may be associated with nutritional and nonnutritional factors. Nonetheless, no prior research has investigated this critical public health issue. Therefore, the current study addresses this knowledge gap by presenting data on the rate of serious BDs and associated factors among LBW newborns. Additionally, our findings are aimed at providing evidence‐based insights to enable the government and nongovernmental stakeholders to design effective interventions for improving maternal and child health in the area.

## 2. Methods

### 2.1. Study Setting

Awi Zone is one of the 12 zones in the Amhara Regional State with a population of over 982,942 [17]. The bureaucratic framework includes nine Woredas and three city administrations with 233 kebeles. This zone features a quartet of state‐run primary and general hospitals, one nonpublic hospital, and numerous clinics. The study was conducted from December 2022 to July 2023 in three of the primary hospitals and one general hospital within this zone. Injibara, Awi Zone′s administrative seat, is located 122 km from Bahir Dar (administrative seat of Amhara Regional State) and 445 km from Addis Ababa (the capital city of Ethiopia).

### 2.2. Study Design

A cross‐sectional design was employed, with data collected across participating hospital settings.

### 2.3. Operational Definition

In Ethiopia health care system, “Primary Hospital” was defined as the first referral level that handles cases from health centers before potential transfer to the general hospitals, with services coverage for 60,000–100,000 people and bed capacity of 30–60 [18]. “General Hospital” was defined as the secondary level referral facilities providing specialized inpatient and emergency care beyond primary hospitals that handle cases from primary hospitals, with services coverage for 1–1.5 million people and bed capacity of 120–300 [19]. A newborn classified as having LBW if the born weight is < 2500 g but ≥ 1500 g (1.5–2.5 kg) and VLBW defined as birth weight < 1500 g but ≥ 1000 g (1–1.5 kg).

### 2.4. Study Variables

#### 2.4.1. Outcome Variable: BD

Independent variables: Sociodemographic characteristics (residency, family monthly income [in birr], ethnicity, religion, marital status, maternal age, maternal occupation, paternal occupation, and maternal level of education), obstetric history and medical backgrounds (medical illness, parity, dietary counsel, additional food intake, birth interval, antenatal care (ANC) visit, gravidity, midupper arm circumference (MUAC), history of miscarriage, type of pregnancy, history of died newborn after 28 weeks gestation, foliate supplementation status, and obstetrics complication), and newborn profile (sex of newborn and range of newborn weight).

### 2.5. Study Population

Over the course of the study, mother–LBW neonate dyads were deemed eligible if the birth took place at either the three primary hospitals or one general hospital, or if home delivery was followed by admission to the aforementioned health facilities within 24 h postpartum.

### 2.6. Sample Size

Given that the total count of LBW births recorded across the four government facilities during the study time frame, random sampling was not feasible. Consequently, all 311 eligible and accessible mother–newborn dyads were enrolled as the study sample.

### 2.7. Inclusion

LBW neonates born at either the three primary hospitals or one general hospital along with home born infants who were admitted to the aforementioned health facilities within 24 h postpartum were considered for the study.

### 2.8. Exclusion

The study excluded the twins with the exception of conjoined twins due to their unique pathophysiological and clinical significance. Furthermore, infants born from mothers with recognized chronic or gestation‐specific health issues.

### 2.9. Data Compilation Processes

We first secured ethical approval from the Research and Publication Office of Injibara University, College of Medicine and Health Sciences, and then delivered signed request letters to the authorities at each study facility, clearly communicating the research purpose and potential gains. Qualified midwives then collected the data using a mix of structured and semistructured interview questionnaires administered to the mothers, alongside clinical data obtained from the newborn′s physical assessments (File S1).

The survey instrument was pilot‐tested on a sample sharing comparable demographic characteristics at similar healthcare facilities prior to its deployment in the target population. Based on the results, the necessary refinements were implemented ahead of primary data collection. To uphold the validity of collected data, experienced midwives with proven proficiency in delivery care and neonatal physical assessment were recruited and provided with prestudy training on data collection techniques. To secure participants′ full cooperation, interviewers were instructed to maintain the privacy and confidentiality of all information obtained. Furthermore, investigators performed random supervisory spot checks during both maternal interviews and neonatal physical examinations.

Upon maternal admission to the maternity ward, data on sociodemographic variables and obstetric history were collected. For newborns delivered in the health facility, weight measurement and congenital anomaly screenings were performed immediately postpartum. For those born at home who were brought to hospital within the first 24 h, the same evaluations were carried out upon their arrival at the facility.

### 2.10. Statistical Methodology and Inference

Before conducting the summary statistics that synthesized obstetrics and neonatal profiles as frequencies and percentages, the entire dataset underwent verification for disparities. Initially gathered via structured and semistructured interviewer administered tools, the data were subsequently validated, edited, coded, and entered into SPSS Version 23. To uncover relationships between outcome and predictor variables, the chi‐square test was executed. Unadjusted logistic regression analysis was then computed to select candidate variables for the adjusted model, pinpointing factors linked to BDs among obstetrics and neonatal profiles. Variables with *p* value ≤ 0.20 from the unadjusted analysis were subsequently entered into the adjusted logistic regression model to find out their distinct impacts on the outcome variable. The odds ratio with a 95% confidence interval (CI) was calculated to evaluate the frequency plus strength of associations, and statistical significance was set at *p* < 0.05.

## 3. Results

### 3.1. Sociodemographic Variables

A 100% response rate was achieved in this study, which enrolled 311 births across three primary hospitals and one general hospital. The majority of participants reside in urban areas (52.1%, *n* = 162) compared with rural areas (47.9%, *n* = 149). Regarding marital status, most women were married (78.5%, *n* = 244), whereas divorced and unmarried women account for 12.9% (*n* = 40) and 8.7% (*n* = 27), respectively. The study sample was characterized by a maternal age distribution where the largest group was aged 26–35 years (37.6%, *n* = 117), followed by those aged ≤ 25 years (31.2%, *n* = 97) and 36–49 years (31.2%, *n* = 97). Educational attainment among mothers was low: 35.7% (*n* = 111) could neither read nor write, 25.7% (*n* = 80) could read and write, 19.6% (*n* = 61) completed primary education, 14.5% (*n* = 45) completed secondary education and only 4.5% (*n* = 14) was college or university graduate. In terms of maternal occupation, farming was most common (44.4%, *n* = 138), followed by government employment (25.7%, *n* = 80), housewife (23.2%, *n* = 72), and merchant (6.8%, *n* = 21). Paternal occupation similarly showed farming as predominant (33.8%, *n* = 105), government employment (27.3%, *n* = 85), daily labor (20.6%, *n* = 64), and merchant (18.3%, *n* = 57) also represented.

Concerning monthly family income among the study subjects, 63.0% (*n* = 196) earned less than 15,000 ETB, 27.3% (*n* = 85) earned between 15,000–25,000 ETB, and only 9.6% (*n* = 30) earned above 25,000 ETB. Majority of the study participants were Orthodox Christians (80.1%, *n* = 249), followed by Muslim (15.8%, *n* = 49), and Protestant (4.2%, *n* = 13). Last but not least, ethnically, the sample was nearly evenly split between Agew (51.8%, *n* = 161) and Amhara (48.2%, *n* = 150) (Table 1).

**Table 1:** Comprehensive sociodemographic indicators of the guardians (parents).

Variables	Frequency	Percent
Residency		
Rural	149	47.9
Urban	162	52.1
Ethnicity		
Amhara	150	48.2
Agew	161	51.8
Religion		
Orthodox	249	80.1
Muslim	49	15.8
Protestant	13	4.2
Marital status		
Married	244	78.5
Unmarried	27	8.7
Divorced	40	12.9
Maternal level of education		
University (college) graduate	14	4.5
Secondary education completed	45	14.5
Primary education completed	61	19.6
Can read and write	80	25.7
Cannot read and write	111	35.7
Maternal occupation		
Housewife	21	6.8
Merchant	72	23.2
Farmer	138	44.4
Government employee	80	25.7
Paternal occupation		
Farmer	105	33.8
Government employee	85	27.3
Merchant	57	18.3
Daily laborer	64	20.6
Family monthly income (in birr)		
< 15000 ETB	196	63.0
15,000–25,000 ETB	85	27.3
> 25,000 ETB	30	9.6
Maternal age category in (years)		
≤ 25 years	97	31.2
26–35 years	117	37.6
36–49 years	97	31.2

### 3.2. Medical Backgrounds and Obstetric History

Pertaining to parity, 18.6% (*n* = 58) had no previous birth (primiparous), 46.3% (*n* = 144) had 2–4 births, and 35.0% (*n* = 109) had five or more births. With respect to birth interval, 16.7% (*n* = 52) were primigravid, 26.0% (*n* = 81) had an interval greater than 3 years, 22.2% (*n* = 69) had an interval of 2–3 years, and 35.0% (*n* = 109) had an interval less than 2 years. Dietary counseling was received by 54.3% (*n* = 169) of the subjects, whereas 45.7% (*n* = 142) did not receive such counseling.

Regarding medical and obstetric profile of the study subjects, the majority (63.3%, *n* = 197) had no ANC visit, whereas 36.7% (*n* = 114) attended at least once. Regarding foliate supplementation during the current pregnancy, 58.5% (*n* = 182) reported taking supplements, whereas 41.5% (*n* = 129) did not. In terms of gravidity, most were multigravid (69.5%, *n* = 216), and 30.5% (*n* = 85*%*) were primigravid. For the type of pregnancy, 28.0% (*n* = 87) reported the pregnancy as unwanted and unintended, 44.7% (*n* = 138) as wanted and intended, and 27.3 (*n* = 85) as wanted but unintended.

Gestational extra dietary consumption was reported by 41.2% (*n* = 128) whereas 58.8% (*n* = 183) did not increase their food intake. A history of newborn death after 28 weeks of gestation was present in 11.9% (*n* = 37) of subjects, and 88.1% (*n* = 274) had no such history. History of miscarriage was reported by 10.6% (*n* = 33), whereas 89.4% (*n* = 278) had no prior miscarriage. Regarding MUAC, 57.9% (*n* = 180) had measurement below 22 cm and 42.1% (*n* = 131) measured 22 cm or above. Medical illness during pregnancy was noted in 14.1% (*n* = 44) of the participants, whereas 85.9% (*n* = 267) had no medical illness. Finally, obstetric complications were experienced by 27.0% (*n* = 84) and 73.0% (*n* = 227) had no such complications (Table 2).

**Table 2:** Obstetric history and medical backgrounds.

Characteristics	Frequency	Percent
ANC visit		
No	114	36.7
Yes	197	63.3
Foliate supplementation current pregnancy		
No	182	58.5
Yes	129	41.5
Gravidity		
Multigravida	216	69.5
Primigravida	85	30.5
Type of pregnancy		
Wanted and intended	87	28.0
Wanted but unintended	139	44.7
Unwanted and unintended	85	27.3
Parity		
≥ 5 births	58	18.6
2–4 births	144	46.3
P0	109	35.0
Birth interval		
> 3 years	52	16.7
2–3 years	81	26.0
< 2 years	69	22.2
Primigravida	109	35.0
Dietary counsel		
No	169	54.3
Yes	142	45.7
Additional food intake		
No	128	41.2
Yes	183	58.8
History of died newborn after 28 weeks gestation		
No	274	88.1
Yes	37	11.9
History of miscarriage		
No	278	89.4
Yes	33	10.6
MUAC		
< 22	131	42.1
≥ 22	180	57.9
Medical illness		
No	267	85.9
Yes	44	14.1
Obstetrics complication		
No	227	73.0
Yes	84	27.0

### 3.3. Neonatal Profile and Frequency of BD

Two hundred fifty‐eight (83%) of the newborns had no overt congenital anomalies whereas 53 (17%) of newborns had at least one kind of BDs. Regarding the investigation of sex of newborns revealed that preponderance 200 (64.3%) were male. A total of 237 (76.2%) newborns had LBW, whereas the rest (74, 23.8%) of newborns had VLBW during delivery (Table 3). Among the newborns identified in this study as having at least one BD, the calculated prevalence rate was 16.1%. Nearly half of these affected infants (51.1.1%) were male. Importantly, all 53 cases presented with major malformations (Table 3).

**Table 3:** Variables pertaining to neonatal profile.

Characteristics	Frequency	Percent
Sex of newborn		
Male	200	64.3
Female	111	35.7
Range newborn weight		
Low birth weight	237	76.2
Very low birth weight	74	23.8
Congenital anomalies		
No	258	83.0
Yes	53	17.0
Congenital anomaly type		
Amelia	11	3.54
Spinal bifida	9	2.89
Club foot	9	2.89
Clift lip with palate	7	2.25
Anencephaly	5	1.6
Gastroschisis	5	1.6
Thoracophagus with clift lip and palate	4	1.28
Massive fusion	3	0.96

### 3.4. Categories of BDs

Table 3 presents the specific types of BDs identified in this study. Among the 311 LBW newborns, the most frequently observed anomaly involved the musculoskeletal system with a prevalence of 6.43% (20 cases). Central nervous system anomalies followed, accounting for 4.50% (14 cases), and orofacial clefts were seen in 2.25% (7 cases). Abdominal wall defect specifically gastroschisis and thoracopagus accompanied by cleft lip and palate contributed 1.60% and 1.28%, respectively. The least common anomaly was massive fusion occurring in only 0.96% of cases. When examining only the newborns with congenital anomalies, musculoskeletal defects remained the most prevalent representing 37.73% of all anomaly cases (Table 3).

**Figure 1:**
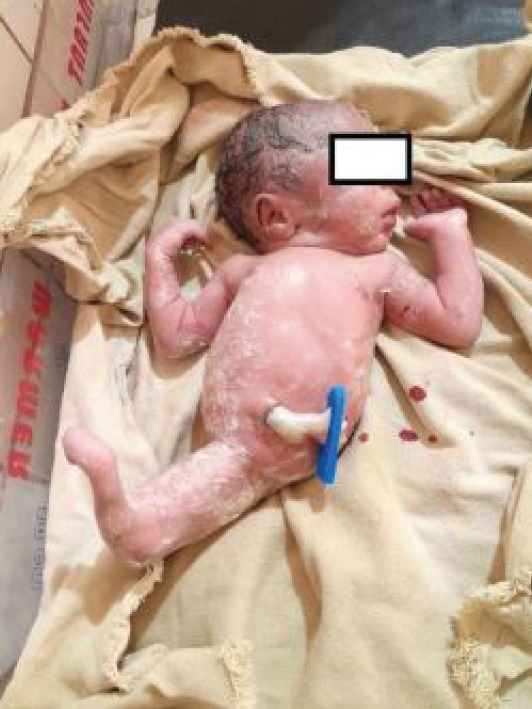
Depiction of neonate with congenital amelia affecting the unilateral lower limb [20].

**Figure 2:**
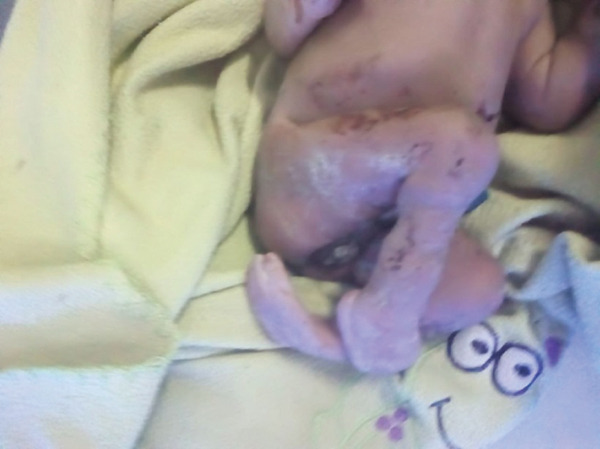
Illustration of bilateral talipes equinovarus (club foot) in neonate.

**Figure 3:**
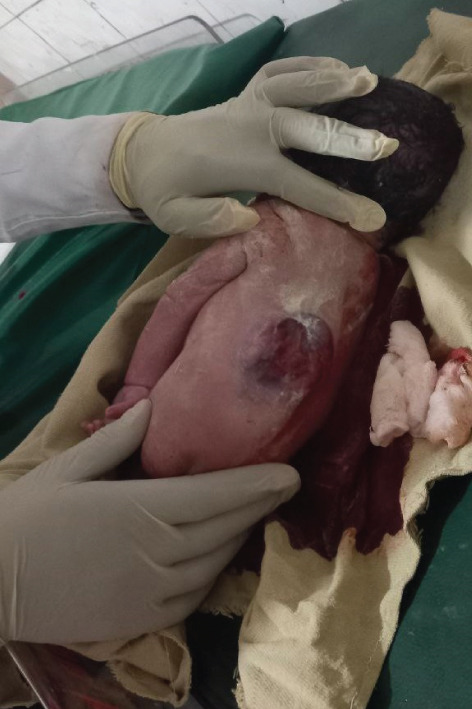
Depiction of spina bifida affecting the thoracolumbar region of neonate.

**Figure 4:**
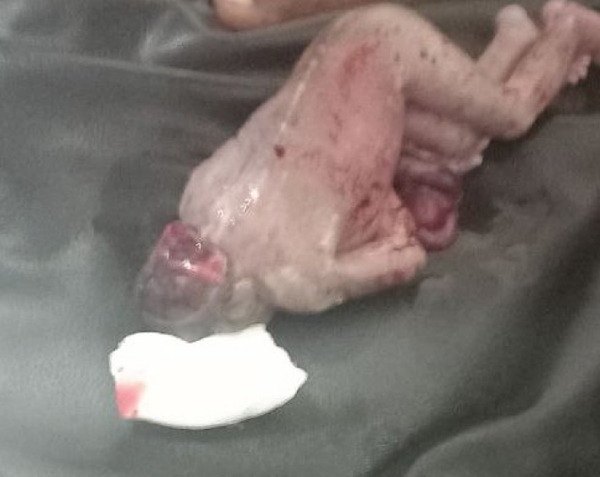
Depiction of an anencephalic neonate, showing absent cranial vault and rudimentary brain tissue.

**Figure 5:**
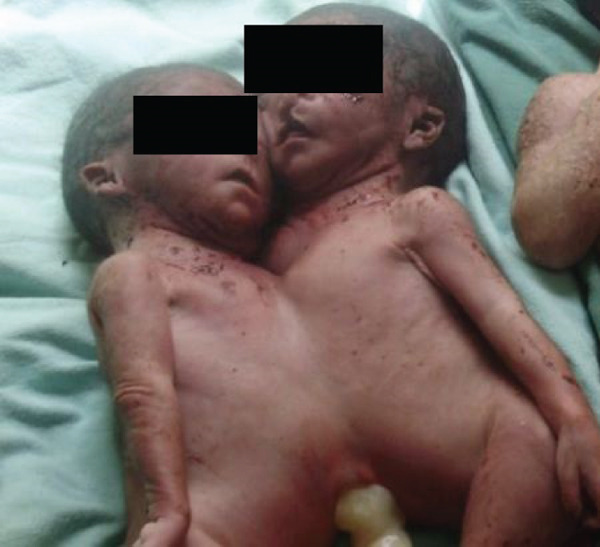
Depiction of congenital thoracic fusion defect with concomitant cleft lip and palate in neonates [20].

**Figure 6:**
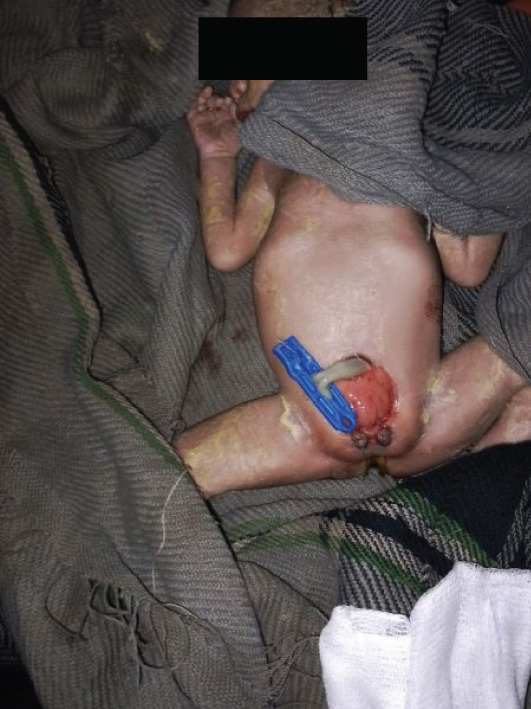
Illustration of neonatal gastroschisis, congenital abdominal wall defect with exposed intestinal loops [20].

**Figure 7:**
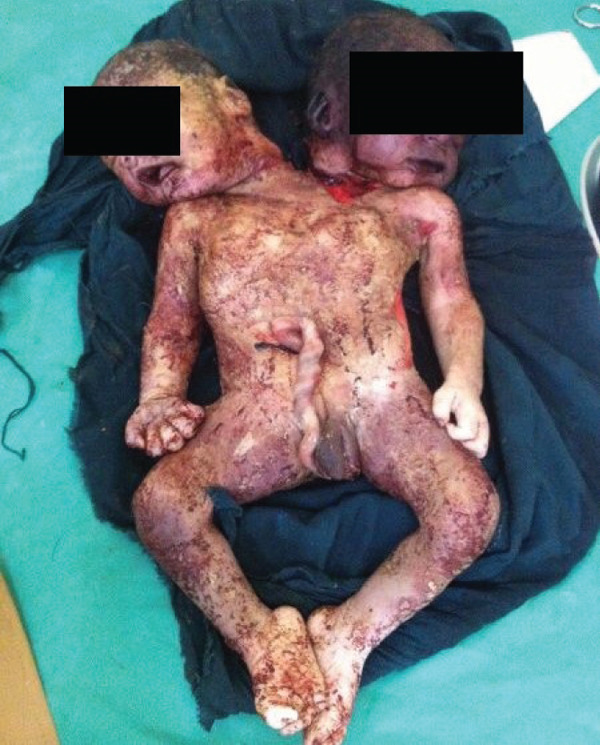
Depiction of extensive thoraco‐omphalopagus fusion in conjoined twins.

Among musculoskeletal malformations (*n* = 20), amelia was the most prevalent (11/20; Table 3 and Figure 1), whereas talipes equinovarus accounted for the remaining 9 cases (Table 3 and Figure 2). Nervous system malformations ranked the second affecting 14 of the 53 neonates; spina bifida was the predominant subtype (9/14; Table 3 and Figure 3) with anencephaly accounting for the other 5 cases (5/14; Table 3 and Figure 4). A total of seven newborns were diagnosed with cleft lip and palate, whereas four newborns presented with thoracopagus combined with cleft lip and palate (Table 3 and Figure 5). Gastroschisis was another anomaly identified in five cases (Table 3 and Figure 6). Massive fusion was the least frequently observed abnormality occurred in three newborns (Table 3 and Figure 7).

### 3.5. Maternal and Perinatal Predictors of BD

The following factors were identified by unadjusted logistic regression analysis as being associated with newborn BDs: extra dietary consumption, history of died newborn before this pregnancy after 28 weeks gestation, foliate supplementation before and during early stage of pregnancy, birth interval, ANC follow‐up, dietary counsel, maternal age, maternal educational status, pregnancy type, and acute medical illness history. After adjustments for potential confounding variables, the following factors remained significantly and independently associated with BDs: maternal age range of 36–49 years, not taking foliate supplements before and throughout early stages of pregnancy, having obstetric complication history, and MUAC < 22 cm.

In comparison with mothers received foliate supplementation, those failed to use it before or throughout the earliest phase of pregnancy faced 4.5 times greater likelihood of delivering infants with BDs (AOR: 4.46; 95% CI: 1.61, 12.40). Maternal MUAC less than 22 (< 22) cm was strongly associated with BD. Those mothers whose MUAC < 22 cm were near to 4 times more likely to give birth with congenital anomalies compared with mothers whose MUAC ≥ 22 cm with (AOR: 3.67; 95% CI: 1.52, 8.83).

Additionally, the likelihood of delivering newborns with congenital anomalies among mothers whose age is between 36 and 49 years old was 3.6 times (AOR: 3.60; 95% CI: 1.26, 10.56) more compared with mothers whose age was less than or equal to 25 years.

The presence of past adverse obstetric events prior to the index pregnancy was found to be significantly correlated with congenital malformations. Epidemiologically, this finding reveals that mothers who had experienced such complications beforehand were 1.9 times more prone to give birth with congenital malformations than those lacking any prior obstetric complication (AOR: 1.90; 95% CI: 0.87, 4.15) (Table 4).

**Table 4:** Predictors of birth defects among LBW newborns in public hospital of Awi Zone, Northwest Ethiopia: hierarchical logistic regression analysis.

Variables	Birth defects		COR (95% CI)	AOR (95% CI)
	No	Yes		
Maternal age				
≤ 25 years	91 (29.3%)	6 (1.9%)	1	1
26–35 years	108 (34.7%)	9 (2.9%)	1.26 (0.43, 3.69)	0.92 (0.27, 3.13)
36–49 years	59 (19.0%)	38 (12.2%)	9.77 (3.89, 24.54)	3.60 (1.26, 10.56) ^∗^
Maternal occupation				
Farmer	116 (37.3%)	22 (7.1%)	1	1
Government employee	76 (24.4%)	4 (1.3%)	0.28 (0.09, 0.84)	0.32 (0.10, 1.09)
Housewife	19 (6.1%)	2 (0.6%)	0.55 (0.12, 2.56)	0.71 (0.12, 4.34)
Merchant	47 (15.1%)	25 (8.0%)	2.81 (1.44, 5.46)	0.98 (0.34, 2.83)
Residence				
Rural	115 (37.0%)	34 (10.9%)	2.23 (1.21, 4.11)	1.15 (0.49, 2.70)
Urban	143 (46.0%)	19 (6.1%)	1	1
MUAC				
<22 cm	92 (29.6%)	39 (12.5%)	5.03 (2.59, 9.74)	3.67 (1.52, 8.83) ^∗∗^
≥22 cm	166 (53.4%)	14 (4.5%)	1	1
History of died newborn after 28 weeks gestation				
Yes	26 (8.4%)	11 (3.5%)	2.34 (1.07, 5.09)	1.39 (0.50, 3.91)
No	232 (74.6%)	42 (13.5%)	1	1
History of miscarriage				
Yes	21 (6.8%)	12 (3.9%)	3.30 (1.51, 7.23)	0.99 (0.36, 2.77)
No	237 (76.2%)	41 (13.2%)	1	1
Gravidity				
Primigravida	91 (29.3%)	4 (1.3%)	1	1
Multigravida	167 (53.7%)	49 (15.8%)	6.68 (2.33, 19.09)	6.46 (0.78, 53.52)
Parity				
P0 (one)	103 (33.1%)	6 (1.9%)		1
2–4 births	120 (38.6%)	24 (7.7%)	3.433 (1.35, 8.72)	0.66 (0.10, 4.42)
≥ 5 births	35 (11.3%)	23 (7.4%)	11.28 (4.25, 29.96)	0.66 (0.09, 5.12)
ANC visit				
Yes	172 (55.3%)	25 (8.0%)	1	1
No	86 (27.7%)	28 (9.0%)	2.24 (1.23, 4.07)	0.62 (0.26, 1.52)
Foliate supplementation				
Yes	123 (39.5%)	6 (1.9%)	1	1
No	135 (43.4%)	47 (15.1%)	7.14 (2.95, 17.28)	4.46 (1.61, 12.40) ^∗∗^
Obstetric complication history				
Yes	58 (18.6%)	26 (8.4%)	3.32 (1.80, 6.13)	1.90 (0.87, 4.15) ^∗^
No	200 (64.3%)	27 (8.7%)	1	1

^∗^ *p* < 0.05,  ^∗∗^ *p* < 0.01.

## 4. Discussion

The current investigation revealed a substantially elevated prevalence of BDs among the cohorts of newly delivered LBW infants and successfully elucidated the crucial associated risk determinants. Regarding the phenotypic distribution, the most frequently encountered malformations were musculoskeletal defects, followed by neural tube defects, orofacial clefts, gastroschisis, thoracopagus complicated by concurrent cleft lip plus palate, and extensive multiorgan fusion deformities.

The observed prevalence estimate from our current study corresponds remarkably well with the findings reported in seminal US investigation, which focus on the high risk neonatal subpopulation weighting under 1500 g, similarly estimated the overall frequency of BDs to be approximately 16%–17%,thereby reinforcing the robustness of our results across geographical distinct settings [21]. LBW and BDs often coexist because defects can restrict fetal growth, whereas factors causing LBW (e.g., poor nutrition and infection) may also disrupt normal organ development [21, 22]. Conversely, the overall burden of BDs ascertained in our study lagged appreciably behind the approximate 20% estimate reported by research teams based in Mexico [14] and United Kingdom [15]. This discrepancy may be stem from alterations in the research plans, scope, settings, and diagnostic criteria employed across the two studies.

In contrast, findings from Xi′an, China (8.59%) [23] and São Paulo, Brazil (4.5%) [24] indicate a comparatively lower prevalence of BDs. This disparity may be explained by differences in social determinants. The presence of conflicts in the Amhara Region of Ethiopia could be another possible explanation for the high prevalence observed in the current research [20].

Compared with neonates whose mothers consumed folic acid during pregnancy, those born to mothers who did not receive supplementation before or during gestation had a 4.46‐fold higher risk of developing BDs. This finding corroborates prior research which demonstrates that ant‐foliate medications increase the incidence of BDs whereas foliate supplementation confers a protective effect [25, 26].

Infants born to mothers with an upper arm circumference less than 22 cm during pregnancy had a 3.67‐fold higher risk of BDs than those born to mothers measuring 22 cm or more. This finding is in line with the scoping review done by Kapil and Ververs [27]. A MUAC less than 22 cm is a robust marker of maternal malnutrition, which can directly or indirectly interfere with the precise process of organogenesis. It does this by creating deficiencies in critical nutrients, increasing oxidative stress, and compromising the placental environment thereby significantly elevating the risk of congenital anomalies in newborns [27, 28].

This study further revealed that mothers aged 36–49 years were 3.6 times more likely to deliver infants with BDs than those aged 25 years or younger. This finding is corroborated by studies conducted in the Metropolitan Atlanta Congenital Defects Program [29], and Newark (United States) [12]. A probable explanation is that females possess a finite quantity of eggs at birth, which are released during each menstrual cycle at ovulation. With advancing age, both the amount plus quality of eggs decline, potentially leading to chromosomal defects that might result in genetic disorders in babies [30].

Lastly in this study, mothers who had a history of obstetric complication before this pregnancy were 1.9 times more prone to have newborns with different congenital anomalies than their counterparts. This finding was supported by a cohort study conducted in Ireland [31]. It is important to clarify that this is often not a direct cause and effect relationship. Instead, a history of complication can be a marker for underlying maternal genetic factors and social determinants [31, 32].

In the preliminary bivariate analysis, both dietary counseling and additional food intake during pregnancy showed significant association with BD outcomes. This association was not sustained in the multivariate analysis after adjusting for potential confounding factors. However, evidence‐based dietary advice and additional food intake during pregnancy are strongly linked to better neonatal outcomes [33]. This investigation revealed that maternal illiteracy alone had no substantial connection to BDs. Yet its real hazard lies in functioning as a potent social health determinant. Illiteracy obstructs access to key health information and pregnancy care, which are critical for diminishing the chances of congenital anomalies [34].

Inability to access ANC service was not associated with BDs in the current study finding, whereas other research confirms it is a significant risk factor and highlights a need for continued focus on early utilization [3]. A potential explanation is that the absence of ANC service itself does not cause BDs; it directly prevents the use of preventive measures (like folic acid). Indirectly serve as powerful marker for constellation of other social, economic, behavioral risk factors that significantly increase the chance of a neonate born with congenital anomalies. Additionally, inability to access and utilize quality ANC in the early stages of pregnancy impedes the control of pre‐existing maternal conditions, including diabetes, epilepsy, and phenylketonuria (PKU), all of which are strongly correlated with an increased incidence of congenital anomalies [32].

## 5. Conclusion and Recommendation

Our findings reveal that 17% of LBW newborns in the study area were diagnosed with BDs. This heightened frequency underscores critical challenges for healthcare providers, etiological researchers, and public health policy makers. Four independent risk factors emerged as significantly associated with BDs: absences of foliate supplementation before and during early pregnancy, maternal MUAC below 22 cm, history of obstetric complications, and advanced maternal age (36–39 years). Notably, the musculoskeletal system was the most commonly implicated. In light of these results, we advocate for intensified efforts to provide pregnant women with adequate dietary support, foliate supplements, and timely plus accessible ANC.

**Nomenclature**
ANC: antenatal care
AOR: adjusted odds ratio
BD: birth defect
CI: confidence interval
LBW: low birth weight
MUAC: midupper arm circumference
SPSS: Statistical Package for Social Science
VLBW: very low birth weight
WHO: World Health Organization

## Funding

No funding was received for this manuscript.

## Ethics Statement

Ethical clearance was acquired from the Research Counsel Member of the College of Medicine and Health Sciences (CMHS), Injibara University with Ethics Approval Number ቁጥር/እን/ዩን/ህ/ጤ/ሳ/ኮ/326/09. Additionally, each data collecting site′s hospitals and the Awi Zone Health Department have granted their consent. Since the data collected in this study were a part of medical services with a routine pattern in hospitals, and the mode of data collection was noninvasive, verbal informed consent was obtained from each study participant′s mother/caretaker before data collection and documented by data collectors. This approach for getting verbal agreement to participate was approved by the Research Counsel Member of CMHS. Confidentiality of the data was protected by omitting their identifiers. All individuals were participating in this study only with their willingness.

## Consent

Publication consent was incorporated into this study′s main informed consent process. All mothers or caretakers were fully informed of the objectives and intent to disseminate aggregated findings in journals, conferences, and institutional reports. All identifiable images included in the manuscript depict deceased neonates captured immediately after delivery and explicit parental permission was obtained for their publication. For all remaining study, participants provided consent under strict confidentiality guarantee that no personal identifiers, such as names, medical records, and residential addresses would appear in any publication.

## Conflicts of Interest

The authors declare no conflicts of interest.

## Supporting information


**Supporting Information** Additional supporting information can be found online in the Supporting Information section. File S1 is pretested, interviewer‐administered questionnaires and checklist used for data collection. The tool was adopted from different literatures developed for similar purposes by different authors.

## Data Availability

The data that support the findings of this study are available on request from the corresponding author. The data are not publicly available due to privacy or ethical restrictions.
