# Supplementary material for: Low Birth Weight Newborns at Public Hospitals in the Awi Zone of Northwest Ethiopia: Birth Defects and Associated Variables
Source: Int J Pediatr. 2026 Aug 8;2026:2312755. doi: 10.1155/ijpe/2312755 (PMC13451932; doi:10.1155/ijpe/2312755)
Supplement: Supplementary file 1 [file IJPE-2026-2312755-s001.docx]

**Consent and questionnaire (English version)**

A questionnaire on the assessing of Birth prevalence and factors associated with birth defect among low birth weight newborns at a public hospital of Awi Zone, Southwestern Ethiopia: Hospital based cross-sectional study, Ethiopia

**Introduction**

My name is	_____________, we are conducting a survey research on birth prevalence and associated factors of congenital anomalies among neonates born at a public hospital in Awi Zone and we are going to collect data to study the above mentioned topic. I would like to tell you that would have a short discussion with you concerning this study. Before we directly go to our discussion, I will request you to listen carefully to what going to be read to you about the purpose & general condition of the study as it will help you decide to participate or not. The study will be conducted through a structure questionnaire completed by the data collectors. We are asking you for a little of your time about 30-40 minute to help in this study. We would like to assure you that the privacy & confidentiality should strictly be secure throughout. All your information will be numbered & your name will not be used, your answer to any of the questions will not be given to any else & not reports to the study will ever identify you. If the report of the result is published only information about the total group will appear. The interview is voluntary your participation/ non- participation or refusal to answer question will not have on effect now or in the future on you are you willing to participate in interview.

Yes----------continue interview

No------------thank you

**Date collection:**

Time started:	Time finished:

Data collector’s name ______________Signature__________ Date	___

Supervisor’s name_________________ Signature _________

#### Thank you for your time and cooperation!!!!!

**For English question**

**Section 01: Socio-Demographic Characteristics of the mother**

| S. No | Question | Response | Code |
| --- | --- | --- | --- |
| 01 | How old are you?(maternal) | ___________years |  |
| 02 | Residence | 1. Rural  2. Urban |  |
| 03 | Religion | 1. Orthodox  2. Muslim  3 .Protestant  4. Catholic  5. Other (specify)_____________ |  |
| 04 | Ethnicity | 1. Amhara  2. Agew  3. Oromo  4. Tigray  5. Other (specify)__________ |  |
| 05 | Marital status | 1. Married  2. Divorced  3. Unmaried  4. Separately exist |  |
| 06 | Maternal level of education | 1. Cannot read and write  2. Can read and write  3. Primary completed  4. Secondary completed  5 . University/ College graduate  6. Other (specify)_______________ |  |
| 07 | Occupation of the pregnant woman | 1. House wife  2. Government employee  3. Merchant  4. Student  5. Daily laborer  6. Farming  7. Other(specify) |  |
| 08 | Occupation of the husband | 1. Government employee  2. Merchant  3. Student  4. Daily laborer  6 Farming  7 Other(specify) |  |
| 09 | Monthly income of family | _____________ in Ethiopian Birr |  |
| 10 | Mid upper arm  Circumference measurement of the mother | …………………centimeter |  |

**Section 02: Maternal Medical and Obstetric Profile**

|  | Questions | Response | Code |
| --- | --- | --- | --- |
| 11 | Number of gravidity | ___________in number |  |
| 12 | Number of parity | ___________in number |  |
| 13 | What is your birth spacing interval in year before this pregnancy (Inter pregnancy interval)? | 1. <1 year 2. <2 - 1years 3. <3 -2 years 4. <4-3 years 5. <5-4 years 6. 5 years and above |  |
| 14 | What is your need of current pregnancy? | 1.Wanted and planned(supported)  2.Wanted but un planned  3.Un wanted and un planned |  |
| 15 | Have you ever experienced any miscarriage? | 1.Yes  2.No |  |
| 16 | Have you ever experienced died newborn before this pregnancy after 28 weeks of gestation? | 1.Yes  2.No |  |
| 17 | Have you obtained dietary counseling by health professionals during recent pregnancy? | 1.Yes  2..No |  |
| 18 | Have you taken additional food during this recent pregnancy? | 1.Yes  2.No |  |
| 19 | Did you have ANC follow up to recent pregnancy? | 1.Yes  2.No |  |
| 20 | Did you take Iron folic acid tablets for recent pregnancy? | 1. Yes 2. No |  |
| 21 | Have you had any acute medical illness history during the recent pregnancy? | 1.Yes  2.No |  |

**Section 03: Variables Related to the neonates of the recent pregnancy**

| S.No | Question | Response | Code |
| --- | --- | --- | --- |
| 22 | Sex of the current new born? | 1.Male  2.Female |  |
| 23 | Weight of new born in grams | …………………….grams |  |
| 24 | Range of the weight of the newborn | 1.Low birth wt (<2500gm-1500 gm)  2.Very low birth wt(<1500-1000gm)  3.Extremely low birth wt (<1000gm) |  |

04. **Information about the presence and types of congenital anomalies**

| **Congenital malformation** | | | |
| --- | --- | --- | --- |
| **25. Are there congenital anomalies** | | 1.Yes  2. No |  |
| **26. If yes, what types of birth defects does the child has?** | |  |  |
| **Central Nervous system**   1. Hydrocephalus 2. Meningomyelocele 3. Microcephaly 4. Anencephaly 5. Encenphocele 6. Craniorachischisis | |  |  |
| **Gastrointestinal defects**   1. Trachea-esophageal fistula 2. gastroschisis 3. Diaphragmatic hernia 4. Duodenal atresia 5. Imperforate anus 6. Ectopic anus 7. Congenital inguinal hernia 8. Umbilical hernia 9. Epigastric hernia | |  |  |
| **Genitourinary defects**   1. Amibigious genitalia 2. Urethral fustula 3. Meatal stenosis 4. Penile tourniquet 5. Hypospadias 6. Epispadias | |  |  |
| **Musculoskeletal defects**   1. Spinal bifida 2. Club foot: bilateral ______ unilateral 3. Ankyloglossia 4. Palatine fistula 5. Clefet lip only: median _,bilateral__, unilateral___ 6. Cleft palate only: median ____,bilateral__, unilateral___ 7. Cleft lip with palate: median ____,bilateral__, unilateral___ 8. Fistula in the neck 9. Congenital torticilis 10. Others:___________________________________ |  | | |

**Thank you very much for sparing time to participate in this study!!!**
